# Effects of *Vitex trifolia* L. leaf extracts and phytoconstituents on cytokine production in human U937 macrophages

**DOI:** 10.1186/s12906-020-02884-w

**Published:** 2020-03-18

**Authors:** Hai-Ning Wee, Soek-Ying Neo, Deepika Singh, Hui-Chuing Yew, Zhi-Yu Qiu, Xin-Rong Cheryl Tsai, Sin-Yi How, Keng-Yan Caleb Yip, Chay-Hoon Tan, Hwee-Ling Koh

**Affiliations:** 1 Department of Pharmacy, Faculty of Science, National University of Singapore, 18 Science Drive 4, Singapore, 117543 Singapore; 2 Department of Pharmacology, Yong Loo Lin School of Medicine, 16 Medical Drive, Block MD3, #04-01S, Singapore, 117600 Singapore

**Keywords:** *Vitex trifolia*, TNF-α, IL-1β, Artemetin, Casticin, Vitexilactone, BHT, Maslinic acid

## Abstract

**Background:**

Dysregulation of pro-inflammatory cytokines such as tumor necrosis factor-α (TNF-α) and interleukin-1β (IL-1β) form the basis of immune-mediated inflammatory diseases. *Vitex trifolia* L. is a medicinal plant growing in countries such as China, India, Australia and Singapore. Its dried ripe fruits are documented in Traditional Chinese Medicine to treat ailments like rhinitis and dizziness. Its leaves are used traditionally to treat inflammation-related conditions like rheumatic pain.

**Objective:**

This study aimed to investigate the effects of *V. trifolia* leaf extracts prepared by different extraction methods (Soxhlet, ultrasonication, and maceration) in various solvents on cytokine production in human U937 macrophages, and identify phytoconstituents from the most active leaf extract.

**Methods:**

Fresh leaves of *V. trifolia* were extracted using Soxhlet, ultrasonication, and maceration in hexane, dichloromethane, methanol, ethanol or water. Each extract was evaluated for its effects on TNF-α and IL-1β cytokine production by enzyme-linked immunosorbent assay in lipopolysaccharide-stimulated human U937 macrophages. The most active extract was analyzed and further purified by different chemical and spectroscopic techniques.

**Results:**

Amongst 14 different leaf extracts investigated, extracts prepared by ultrasonication in dichloromethane and maceration in ethanol were most active in inhibiting TNF-α and IL-1β production in human U937 macrophages. Further purification led to the isolation of artemetin, casticin, vitexilactone and maslinic acid, and their effects on TNF-α and IL-1β production were evaluated. We report for the first time that artemetin suppressed TNF-α and IL-1β production. Gas chromatography-mass spectrometry analyses revealed the presence of eight other compounds. To the best of our knowledge, this is the first report of butylated hydroxytoluene, 2,4-di-*tert*-butylphenol, campesterol and maslinic acid in *V. trifolia* leaf extracts.

**Conclusions:**

In conclusion, leaf extracts of *V. trifolia* obtained using different solvents and extraction methods were successfully investigated for their effects on cytokine production in human U937 macrophages. The findings provide scientific evidence for the traditional use of *V. trifolia* leaves (a sustainable resource) and highlight the importance of conservation of medicinal plants as resources for drug discovery. Our results together with others suggest further investigation on *V. trifolia* and constituents to develop novel treatment strategies in immune-mediated inflammatory conditions is warranted.

## Background

Inflammation, the body’s natural defence mechanism against injury and infection, is harmful when excessive. Chronic inflammation is known as the driving force of the pathogenesis of immune-mediated inflammatory diseases such as inflammatory bowel disease, rheumatoid arthritis and psoriasis [1]. Although distinct in their clinical manifestations, these immune-mediated inflammatory diseases share common pathogenesis pathways, one of which is the dysregulation of cytokines, for example the overproduction of pro-inflammatory cytokines TNF-α and IL-1β [2, 3]. Current treatment approaches of inflammation include the use of non-steroidal anti-inflammatory drugs and glucocorticoids, as well as novel biologic agents that target specific molecules such as TNF-α and IL-1β [4–6]. However, long-term use of existing repertoire of drugs is accompanied with risks of serious adverse side effects, including opportunistic infections, malignancies, anaphylactic reactions, iatrogenic Cushing’s syndrome and osteroporosis [5]. Moreover, novel biologic agents targeting specific molecules though effective come with high costs. Hence, the need to discover alternative anti-inflammatory compounds remains relevant.

Medicinal plants have been documented to treat diverse human ailments for thousands of years and offer a rich resource of novel therapeutics. *Vitex trifolia* L. var. trifolia (*V. trifolia*) from the Verbenaceae family is a deciduous plant mainly found in the coastal areas of Pacific-Asian regions including countries such as China, India, Australia and Singapore [7]. Commonly known as Panikisanbhalu (Hindi), three-leaf chaste tree (English) and 三叶蔓荆 (Chinese), *V. trifolia* is traditionally used for various inflammatory ailments. Dried ripe fruits of *V. trifolia* (also known as *Fructus viticis*) are well documented in Traditional Chinese Medicine to treat ailments like inflammation of the eye, headache, blurred vision, rhinitis, and common cold [8, 9]. Leaves *of V. trifolia* are also used in traditional medicine to treat inflammatory conditions, such as ciguatera fish poisoning in the Pacific region [10]. The leaves are traditionally made into decoction for oral inflammation, or externally applied as a poultice for rheumatic pain and sprains [11]. The flowers are administered orally as infusion for treating intermittent fever accompanied by vomiting and thirst, while the stems are used for dysentery [11, 12]. The roots are used as antiemetic, expectorant and believed to help relieve fever [13, 14]. Several phytochemicals reported in the leaf extracts of *V. trifolia* include flavonoids, such as casticin [15], vitexin [16] and luteolin [17], and terpenes such as eucalyptol and caryophyllene [18]. A number of studies have been published on the anti-inflammatory effects of *V. trifolia* leaf extracts using various rat experimental models, such as Carrageenan induced paw edema rat model [19–22], and RAW264.7 mouse cell lines induced with lipopolysaccharide [10, 11, 23]. These studies focused on investigating aqueous and ethanol leaf extracts prepared using decoction, Soxhlet or maceration. There is limited information on the different extraction methods and solvents on *V. trifolia*, as well as on its inflammatory activity in human macrophages. Human monocytic cell lines such as U937 differentiated with inflammatory stimuli like phorbol 12-myristate 13-acetate (PMA) offer a model for studying macrophage function [24–26]. We have previously reported in an ethnobotanical survey that *V. trifolia* was one of the fresh medicinal plants commonly used in Singapore [27] and leaf extracts of *V. trifolia* prepared by different extraction methods and solvents exhibited promising anti-proliferative activity in multiple cancer cell lines [28]. This study aimed to investigate the effects of various *V. trifolia* leaf extracts prepared by different extraction methods namely Soxhlet, ultrasonication, and maceration in various solvents, on cytokine production in PMA-differentiated U937 macrophages, and to isolate and identify phytoconstituents from the most active leaf extract.

## Methods

### Reagents

Analytical grade solvents (acetone, dichloromethane, ethanol, ethyl acetate, hexane and methanol) and HPLC-grade methanol and acetonitrile were purchased from Tedia (Fairfield, USA). Water was processed by Milli-Q filter (Millipore Corporation, Billerica, USA). Phorbol 12-myristate 13-acetate (PMA) and lipopolysaccharide (LPS) were from Sigma-Aldrich (USA), dexamethasone, dimethyl sulfoxide (DMSO) were from Sigma-Aldrich (USA), Merck (USA), Hospira (Australia) and MP Biomedical Inc. (USA) respectively. Chemical standards for artemetin, casticin and vitexilactone were from ChemFaces (China), while α-amyrin, β-amyrin, butylated hydroxytoluene (BHT), campesterol, 2,4-Di-*tert*-butylphenol, maslinic acid, phytol, β-sitosterol**,** and stigmasterol were from Sigma-Aldrich (USA).

### Plant source and preparation of plant extracts

Fresh, healthy and mature leaves of *V. trifolia* were harvested from Singapore (Leeward Pacific Pte. Ltd) for extraction. A voucher specimen of *V. trifolia* (VT-0101) was deposited at the Department of Pharmacy Herbarium in National University of Singapore. The plant was identified by Mr. Lua Hock Keong from National Parks Board and by checking with The Plant List [29] and identified with reference to the “World Checklist of Selected Plant Families” [30]. Leaves were washed, air dried and blended using a dry grinder, and extracted using Soxhlet, ultrasonication or maceration in hexane, dichloromethane, 70% *v*/v methanol, 70% *v*/v ethanol and water. The extracts were concentrated under vacuum and stored at 25 °C.

### Isolation of chemical constituents from *V. trifolia* leaf extracts

The dried maceration ethanol crude leaf extract was dissolved in water and partitioned with *n*-hexane, dichloromethane, and butanol; these fractions were analyzed for their effects on cell viability and cytokine production. The resultant dichloromethane fraction was then subjected to column chromatography over silica gel 60 using hexane, dichloromethane and methanol to give sub-purified fraction A1, which was also analyzed for its effects on cell viability and cytokine production.

The ultrasonication dichloromethane crude leaf extract was subjected to column chromatography over silica gel 60 using hexane, dichloromethane and methanol to yield various fractions. One fraction was subjected to semi-preparative HPLC (Agilent 1260 Series HPLC System, Agilent Technologies; ZORBAX SB-C18 column (5 μm, 9.4 × 250 mm; flow rate, 2 ml/min; temperature, 25 °C; detection, UV absorption at 254 nm). For the mobile phase, an initial gradient of 5% acetonitrile and 95% water was set and increased to 20% acetonitrile and 80% water for 5 min, followed by 40% acetonitrile and 60% water for 5 min, and then to 100% acetonitrile for 20 min and finally held at 100% acetonitrile for 15 min to isolate casticin, artemetin and vitexilactone. Maslinic acid was purified on silica gel column using dichloromethane:methanol (99:1) as elution solvent.

### Chemical analyses using gas chromatography-mass spectrometry (GC-MS), nuclear magnetic resonance (NMR) spectroscopy, and liquid chromatography-mass spectrometry (LC-MS)

Concentrations of 5 mg/mL for leaf extract or fractions, or 0.1 mg/mL for commercial standards, were prepared and 1 μL was injected into the GC-MS (Agilent 7890 gas chromatograph with 5975C MSD, USA) with splitless injection mode at 230 °C. An Agilent DB-5MS column (30 m × 0.025 μm, 0.25 mm internal diameter, 0.25 μm film thickness) was used with helium carrier gas. The extracts and fractions were analyzed with a temperature program of an initial temperature of 80 °C for 6 min and increased at a rate of 2 °C/min to 280 °C, which was maintained for 10 min before the run ended. Compounds separated from GC-MS were identified using National Institute of Standards and Technology (NIST) Mass Spectral library versions 27 and 247 (NIST, USA) and Wiley Mass Spectral Database Version 7 (John Wiley & Sons, USA).

The NMR spectra were obtained using a Bruker DRX 400 MHz spectrometer ^1^H at 400 MHz; ^13^C at 100 MHz (Fallanden, Switzerland) in deuterated chloroform and tetramethylsilane as an internal reference.

LC-MS of isolated compounds and standards at 0.1 mg/mL were performed using a 2000 QTRAP (Applied Biosystems, Foster City, USA). Electrospray ionization mass spectra (ESI-MS) were recorded in positive and negative modes. The HPLC (Agilent-1100 LC Binary, Santa Clara, USA) was programmed with an isocratic gradient solvent system of 98% acetonitrile and 2% water, and a flow rate of 200 μL/min for 5 min. The mass-per-charge ratios (*m/z*) scanned ranged from 50 to 1000.

### General cell culture

The human monocytic cell line U937 (CRL-1593.2; ATCC) was cultured in RPMI-1640 medium (ThermoScientific, USA) supplemented with 10% v/v heat-inactivated fetal bovine serum (ThermoScientific, USA) in 5% v/v CO_2_ incubator at 37 °C in a humidified atmosphere.

For macrophage differentiation, U937 cells grown in in RPMI-1640 medium supplemented with 2% v/v fetal bovine serum were treated with 5 ng/mL PMA for 24 h, after which the cells were washed with PBS [24–26]. It is well known that excessive use of PMA in differentiating the cells could induce genetic over-expression and this may potentially mask the effects induced by plant extracts. We have looked at the effect of different PMA concentrations selected from published literature [24–26] in the presence and absence of LPS stimulation on cytokine production, and we found that PMA at 5 ng/mL was able to ensure optimal monocyte differentiation and induce cytokine production. These PMA-differentiated U937 macrophages were also referred to as U937 macrophages in this study.

The leaf extracts and chemical standards were dissolved in DMSO and diluted to the desired concentration before addition to cells. The concentration of DMSO in these dilutions was restricted to no more than 0.4% to minimize potential effects of the solvent on cell growth. To examine the effect of leaf extract, fraction, or standard compound, U937 macrophages were incubated for 6 h with the appropriate agent, and then activated with 50 ng/mL LPS overnight. For investigation of BHT effect on cytokine production, U937 macrophages were incubated with BHT for 6 h. To examine the effect of MCC950 or sulfasalazine on BHT-induced cytokine level, U937 macrophages were incubated with MCC950 or sulfasalazine for 24 h, washed with PBS, and incubated with BHT for 6 h. Cell supernatant was collected and measured for the cytokine level by enzyme-linked immunosorbent assay (ELISA).

### Determination of cell viability by WST-1 assay

Exponentially growing cells were plated in 96-well plates at 3 × 10^4^ cells/100 μL in RPMI medium supplemented with 2% v/v fetal bovine serum, treated with PMA for 24 h to differentiate into macrophages and washed with PBS. These differentiated U937 macrophage cells were treated with the appropriate agent (either crude extract, fraction, compound, or DMSO) for 48 h. Untreated differentiated cells were used as controls. After 48 h, the media was aspirated and replaced with 10% *v/v* WST-1 (Roche, Switzerland) for 1 h. The formazan dye produced was quantified at 440 nm against a reference wavelength of 650 nm using a microplate reader (Tecan infinite M200 PRO, Switzerland). Cell viability was expressed as a percentage of the control cells. The IC_50_ value from cell viability assay was used as a parameter for anti-proliferative potency [31–33] while the IC_20_ value was taken as an indicator for non-toxic dose of test sample [32, 34]. The IC_50_ and IC_20_ values were determined using GraphPad Prism 5 (GraphPad Software, Inc., USA). The results were generated from three independent experiments and each experiment was performed in 5 replicates.

### Evaluation of cytokine production by ELISA assay

Cells were plated in 6-well plates (Costar, USA) at 1 × 10^6^ cells per well in RPMI medium supplemented with 2% fetal bovine serum, treated with PMA for 24 h to differentiate into macrophages and washed with PBS. These differentiated cells were incubated with the appropriate extracts, fractions or compounds as described above. Cell supernatant was collected and analysed for cytokine level using a two-site sandwich ELISA kit from Quantikine (Minneapolis, USA) according to manufacturer’s instructions. Briefly, standards and samples were added to wells pre-coated with antibodies for 2 h, washed, and incubated with cytokine conjugate for 1 h. After washing, substrate solution was added for 20 min, followed by stop solution. The cytokine level present was quantified at 450 nm against a reference wavelength of 540 nm using a microplate reader (Tecan infinite M200 PRO, Switzerland) and absolute concentrations of cytokines were interpolated from their respective standard curves. Standard curves were achieved using standard concentrations of the human IL-1β and TNF-α based on instructions in the Quantikine (Minneopolis, USA) kits. The results were generated from three independent experiments.

### Statistical analysis

Statistical analyses were performed by Statistical Package for the Social Sciences (International Business Machines Corporation, USA). Welch Analysis of Variances (ANOVA) followed by Games-Howell post-hoc test were used. A *p* value < 0.05 was considered significant (denoted as *).

## Results

### Evaluation of *V. trifolia* crude leaf extracts for cytokine production

Crude leaf extracts of *V. trifolia* prepared by Soxhlet, ultrasonication, and maceration in various solvents (hexane, dichloromethane, methanol, ethanol, or water) were first evaluated for their potential cytotoxicity in PMA-differentiated U937 macrophages using WST-1 cell viability assay. Visually, unstimulated U937 cells grew as single cell suspension, while PMA-treated U937 cells adhered tightly to the plastic culture plate, showed some cellular aggregation and appeared macrophage-like. The mean IC_20_ and IC_50_ values of the crude leaf extracts, which refer to the concentrations of extracts required to inhibit 20 and 50% growth of the differentiated cells respectively, are shown in Table 1. Generally, the IC_50_ values of methanol, ethanol and water leaf extracts were relatively higher than those of hexane and dichloromethane leaf extracts, regardless of extraction method. Among the different extraction methods using methanol, ethanol and water as solvents, maceration methanol leaf extract showed the smallest IC_50_ of 84.8 ± 6.6 μg/mL, while Soxhlet water leaf extract exhibited the largest IC_50_ value of 684.5 ± 99.0 μg/mL (Table 1). Among the different extraction methods using hexane and dichloromethane as solvents, ultrasonication dichloromethane leaf extract showed the smallest IC_50_ of 3.2 ± 0.1 μg/mL while Soxhlet dichloromethane extract displayed the largest IC_50_ of 47.6 ± 0.7 μg/mL (Table 1).

**Table 1:** Effects of *V trifolia* crude leaf extracts on the cell viability of U937 macrophages measured by WST-1 cell viability assay

Extraction method	Solvents	IC_**20**_ (μg/mL)	IC_**50**_ (μg/mL)
**Soxhlet**	Hexane	3.0 ± 0.4	5.2 ± 0.5
	Dichloromethane	38.9 ± 0.9	47.6 ± 0.7
	Methanol	111.1 ± 23.1	145.1 ± 13.7
	Ethanol	89.0 ± 25.8	132.5 ± 32.8
	Water	430.6 ± 95.9	684.5 ± 99.0
**Ultrasonication**	Hexane	9.7 ± 1.4	12.2 ± 2.5
	Dichloromethane	2.6 ± 0.3	3.2 ± 0.1
	Methanol	100.9 ± 4.8	120.3 ± 12.0
	Ethanol	159.3 ± 20.3	185.5 ± 33.3
	Water	127.0 ± 27.8	172.1 ± 26.4
**Maceration**	Hexane	5.1 ± 0.8	6.4 ± 0.2
	Dichloromethane	3.0 ± 0.1	4.1 ± 0.5
	Methanol	74.0 ± 10.2	84.8 ± 6.6
	Ethanol	85.9 ± 5.6	101.1 ± 11.7

The IC_20_ and IC_50_ values presented are mean ± SD of 3 independent experiments performed in 5 replicates

**Fig. 1:**
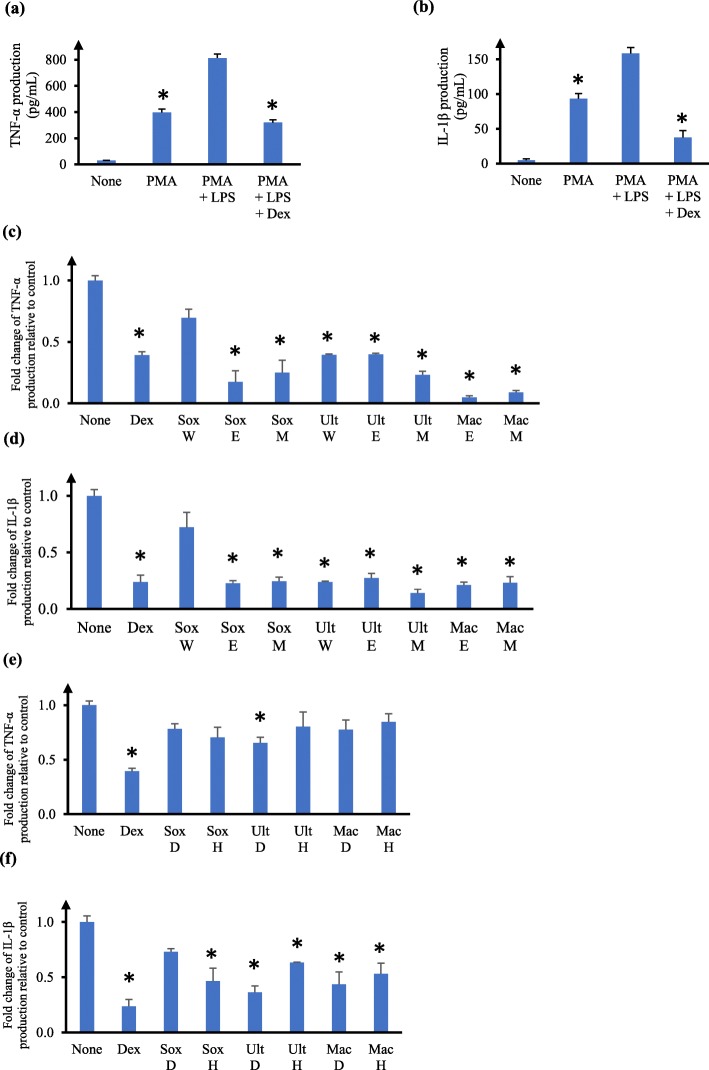
Effects of *V. trifolia* crude leaf extracts on TNF-α and IL-1β production by human U937 macrophages. **a**, **b** Absolute production of TNF-α (**a**) and (**b**) IL-1β by human U937 cells in the presence or absence of PMA, LPS and dexamethasone. **c-f** Fold change of (**c, e)** TNF-α and (**d**, **f**) IL-1β production relative to control in the supernatant of human U937 macrophages. Cells were pre-incubated for 6 h with 100 μg/mL (**c**, **d**) or 2 μg/mL (**e**, **f**) of the appropriate crude leaf extract, followed by LPS stimulation. Sox, Soxhlet; Ult, ultrasonication; Mac, maceration; W, water; E, ethanol; M, methanol; D, dichloromethane; H, hexane. Cytokine production by cells not treated with any extract (ie. none) was taken as 1. Dexamethasone (Dex) at 64.4 ng/mL was used as positive control. The data are presented as mean fold change ± SD of three independent experiments performed in duplicates. *p < 0.05

Figure 1 shows in the absence of any treatment, U937 cells produced very low levels of TNF-α and IL-1β (Fig. 1a and b). Treatment with PMA significantly elevated the production of TNF-α and IL-1β in U937 macrophages. Stimulation of PMA-differentiated cells (also referred here as U937 macrophages) with LPS further doubled the production of both TNF-α and IL-1β compared to PMA treatment only (*p* < 0.05) (Fig. 1a and b). These levels of cytokine secretion are consistent to published reports using U937 as a model system [24–26]. As expected, the increased TNF-α and IL-1β levels were abolished upon pre-incubation of cells with dexamethasone (*p* < 0.05) (Fig. 1a and b), a corticosteroid known to alleviate inflammatory conditions. A criterion for investigating the inflammatory effects of the leaf extracts was that the concentration of leaf extracts used should be the highest one in which the cells remained viable. We chose to use the IC_20_ values of the extracts [32–34], which represented the concentration at which at least 80% of the cell population was alive. Based on Table 1 and our observations of cells treated with different concentrations of extracts, each extract was assayed at 100 μg/mL (for methanol, ethanol and water extracts) or 2 μg/mL (for hexane and dichloromethane extracts) to determine the levels of TNF-α and IL-1β in the supernatant by ELISA.

We first studied the methanol, ethanol and water leaf extracts prepared by Soxhlet, ultrasonication, or maceration on cytokine levels in U937 macrophages. Pre-treatment of U937 macrophages with all except Soxhlet water leaf extract significantly inhibited the production of TNF-α and IL-1β by 60–80% compared to untreated macrophages (*p* < 0.05) (Fig. 1c and d). Maceration ethanol leaf extract was overall most effective in suppressing TNF-α and IL-1β production in U937 macrophages amongst the extracts investigated, while Soxhlet water leaf extract was least effective (Fig. 1c and d).

We next studied the hexane and dichloromethane leaf extracts prepared by Soxhlet, ultrasonication, or maceration. Pre-treatment of U937 macrophages with the ultrasonication dichloromethane leaf extracts significantly inhibited TNF-α production by 40% compared to untreated macrophages (*p* < 0.05), while the other leaf extracts did not significantly affect TNF-α level at the concentration tested (Fig. 1e). Pre-treatment of U937 macrophages with all except Soxhlet dichloromethane leaf extract significantly inhibited IL-1β production by 40–80% compared to untreated macrophages (*p* < 0.05) (Fig. 1f). Ultrasonication dichloromethane leaf extract was overall most effective in suppressing TNF-α and IL-1β levels amongst these extracts, while Soxhlet dichloromethane leaf extract was least effective (Fig. 1e and f).

**Fig. 2:**
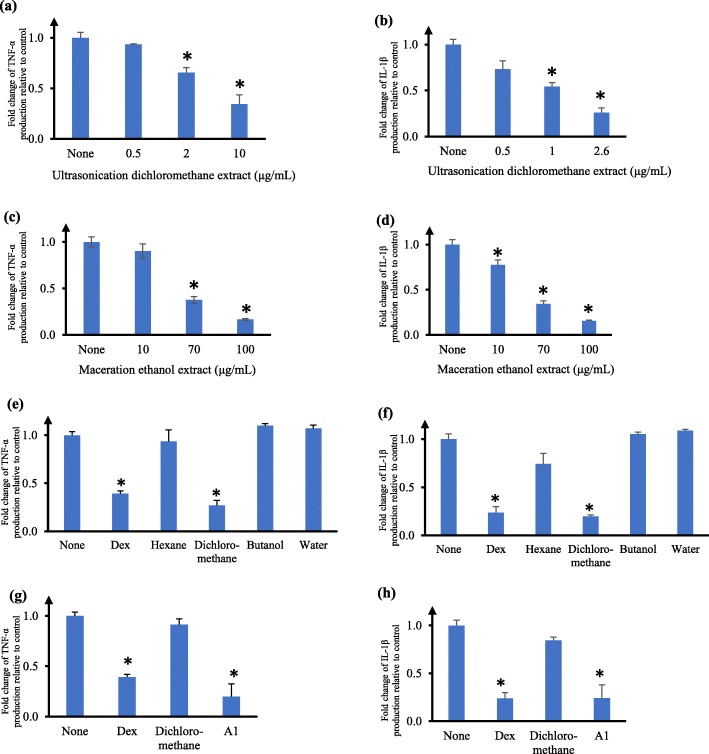
Effects of crude leaf extracts and purified fractions of *V. trifolia* on TNF-α and IL-1β production by human U937 macrophages. Fold change of TNF-α (**a**, **c**, **e**, **g**) and IL-1β (**b**, **d**, **f**, **h**) production relative to control in the supernatant of human U937 macrophages measured by ELISA. Cells were pre-incubated for 6 h with the appropriate extracts or fractions (10 μg/mL in (**e**, **f**); 2 μg/mL in (**g**, **h**)), followed by LPS stimulation. Fractions in (**e**, **f**) were purified from maceration ethanol crude extract, while A1 fraction was purified from the dichloromethane fraction in (**e**, **f**). Cytokine production by cells not treated with any extract or fraction (ie. none) was taken as 1. Dexamethasone (Dex) at 64.4 ng/mL was used as a positive control. The data are presented as mean fold change ± SD of three independent experiments performed in duplicates. *p < 0.05

We further examined the effects of varying concentrations of ultrasonication dichloromethane leaf extract and maceration ethanol leaf extract on cytokine production. The ultrasonication dichloromethane leaf extract reduced TNF-α and IL-1β levels in a concentration-dependent manner, with much smaller IC_50_ values of 4.7 ± 0.9 μg/mL and 1.2 ± 0.2 μg/mL respectively (Fig. 2a and b). The maceration ethanol leaf extract also inhibited TNF-α and IL-1β level in a concentration-dependent manner, with IC_50_ values of 43.6 ± 3.9 μg/mL and 29.2 ± 2.3 μg/mL respectively (Fig. 2c and d). Given the effectiveness of both maceration ethanol and ultrasonication dichloromethane leaf extracts, we chose both extracts for further investigation to identify the active phytoconstituents.

### Isolation and identification of compounds in *V. trifolia* leaf extracts

**Fig. 3:**
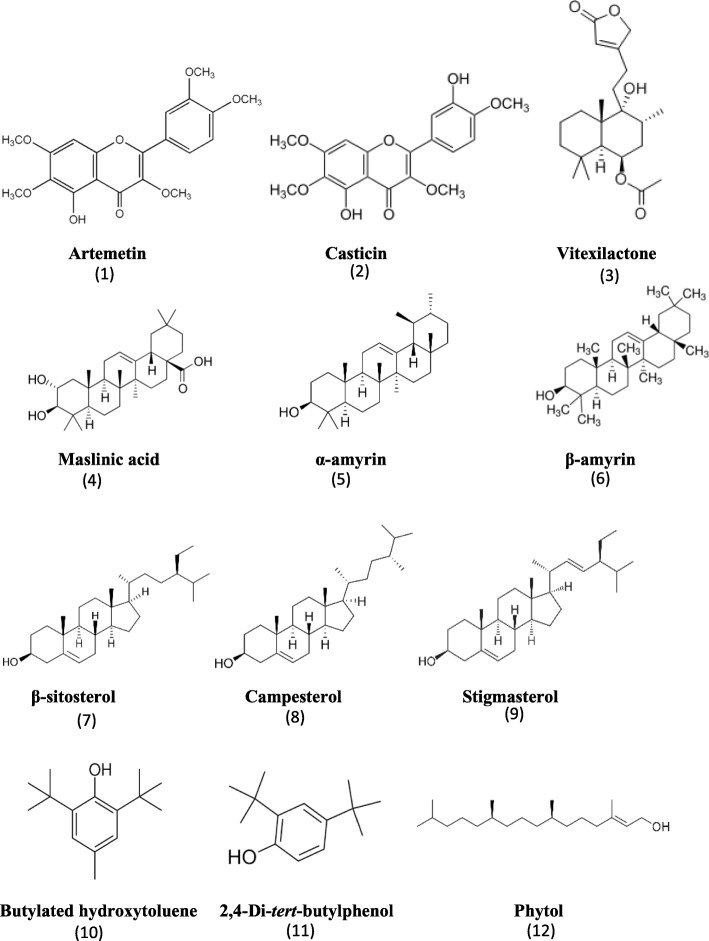
Chemical structures of compounds identified in *V. trifolia* leaves

Gas chromatography-mass spectrometry (GC-MS) analysis was performed on both *V. trifolia* maceration ethanol and ultrasonication dichloromethane crude leaf extracts to characterise their components. Preliminary identification of each compound was based on comparing the mass spectrometric data with the NIST and WILEY reference libraries. The identities of eight putative compounds were confirmed by comparison with the mass spectrometric data of commercial standards, and they are: butylated hydroxytoluene (BHT), 2,4-di-*tert*-butylphenol, phytol, campesterol, stigmasterol, β-sitosterol**,** β-amyrin, and α-amyrin (Fig. 3).

To investigate the active constituents responsible for TNF-α and IL-1β inhibition observed, maceration ethanol crude leaf extract was partitioned using hexane, dichloromethane, n-butanol and water. These fractions were evaluated for their effect on cell viability in U937 macrophages and the results are presented in Table 2**.** Among these four fractions, dicloromethane fraction showed the smallest IC_50_ of 21.1 ± 1.0 μg/mL, while water fraction displayed the largest IC_50_ of 3900.0 ± 450.0 μg/mL (Table 2). The IC_50_ for dicloromethane fraction (21.1 ± 1.0 μg/mL) was about four times less than the IC_50_ for maceration ethanol crude leaf extract (84.8 ± 6.6 μg/mL). We next evaluated the effect of pre-treatment of U937 macrophages with these fractions on TNF-α and IL-1β levels, and found that dichloromethane fraction significantly suppressed TNF-α and IL-1β levels by 80% compared to untreated cells (*p* < 0.05), whereas the other fractions did not show any appreciable difference (Fig. 2e and f).

**Table 2:** Effects of fraction derived from *V. trifolia* maceration ethanol crude leaf extracts on the cell viability of U937 macrophages

Fractions	IC_**20**_ (μg/mL)	IC_**50**_ (μg/mL)
**Hexane**	31.1 ± 8.3	38.1 ± 12.2
**Dichloromethane**	12.9 ± 1.1	21.1 ± 1.0
**Butanol**	139.0 ± 11.1	205.0 ± 16.1
**Water**	2190.0 ± 230.0	3900.0 ± 450.0

The IC_20_ and IC_50_ values presented are mean ± SD of 3 independent experiments performed in 5 replicates

Purification of dichloromethane fraction over column chromatography yielded a sub-purified fraction A1 that, when tested for its effect on cell viability in U937 macrophages, gave an IC_50_ of 6.7 ± 0.1 μg/mL, This is about three times lower than IC_50_ of dichloromethane fraction (21.1 ± 1.0 μg/mL). The sub-purified fraction A1 at 2 μg/ml significantly suppressed the production of TNF-α and IL-1β by almost 80% (*p* < 0.05) (Fig. 2g and h). In contrast, the dichloromethane fraction at 2 μg/ml showed no observable effect on TNF-α and IL-1β production (Fig. 2g and h). Interestingly, this sub-purified fraction A1 was similar in activity as the ultrasonication dichloromethane crude leaf extract, in terms of its effects on TNF-α and IL-1β production and cytotoxicity in U937 macrophages. Since the ultrasonication dichloromethane crude leaf extract had comparatively more yield, we chose it for subsequent studies.

Further purification of the ultrasonication dichloromethane crude leaf extract led to the isolation and identification of casticin (or vitexicarpin), artemetin, vitexilactone, and maslinic acid (Fig. 3). The chemical structures of these isolated compounds were confirmed by comparing NMR and LC-MS data with published data and their respective commercial standards [35–38]. Taken together, 12 compounds were identified in *V. trifolia* leaf extract. To the best of our knowledge, this is the first report of BHT, 2,4-di-*tert*-butylphenol, campesterol and maslinic acid in leaf extracts of *V. trifolia*.

### Evaluation of compounds for cell viability and cytokine production in U937 macrophages

Artemetin, casticin, vitexilactone, maslinic acid and BHT were first investigated for their effects on cell viability in U937 macrophages. Artemetin, maslinic acid and BHT inhibited the growth of these cells with IC_50_ values 125.6 ± 15.3 μg/mL (323.4 ± 39.3 μM), 108.8 ± 4.7 μg/mL (230.2 ± 9.9 μM), and 17.0 ± 0.2 μg/mL (77.1 ± 0.9 μM) respectively. The IC_20_ values were: artemetin 109.5 ± 14.1 μg/mL (281.9 ± 36.3 μM), maslinic acid 64.6 ± 3.0 μg/mL (136.7 ± 6.3 μM), and BHT 14.0 ± 1.5 μg/mL (63.5 ± 6.8 μM). Casticin and vitexilactone did not show any appreciable effect on cell viability of U937 macrophages up to concentrations of 200 μg/mL.

**Fig. 4:**
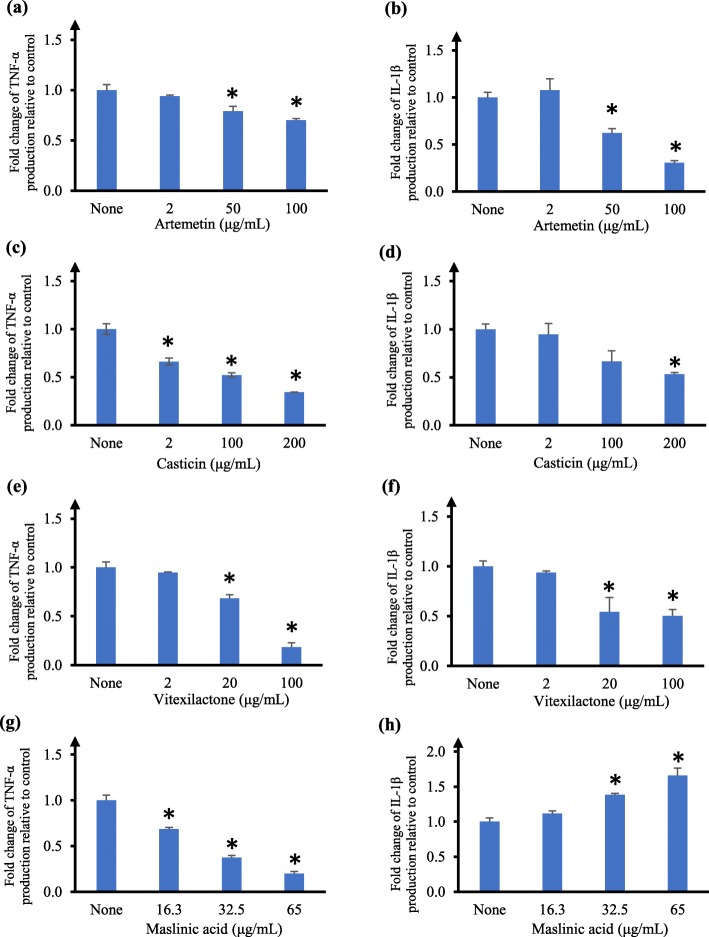
Effects of artemetin, casticin, vitexilactone and maslinic acid on TNF-α and IL-1β production by human U937 macrophages**.** Fold change of TNF-α (**a**, **c**, **e**, **g**) and IL-1β (**b**, **d**, **f**, **h**) production relative to control in the supernatant of human U937 macrophages measured by ELISA. Cells were treated with none or varying concentrations of artemetin (**a**, **b**), casticin (**c**, **d**), vitexilactone (**e**, **f**), and maslinic acid (**g**, **h**) for 6 h, followed by LPS stimulation. Cytokine production by cells not treated with compound (ie. none) was taken as 1. The data are presented as mean fold change ± SD of three independent experiments performed in duplicates. *p < 0.05

We next evaluated these compounds for their effects on TNF-α and IL-1β production in U937 macrophages. Artemetin at 50 μg/mL and 100 μg/mL reduced TNF-α level to 20 and 30% respectively (Fig. 4a), and its IC_50_ value of inhibitory effect on TNF-α could not be determined at the highest concentration of 100 μg/mL achievable. The reduction in TNF-α levels by artemetin at 50 μg/mL and 100 μg/mL were not due to decrease in cell viability as we did not observe any significant difference in cell viability at the same concentrations. Artemetin at 50 μg/mL significantly reduced IL-1β level to 60% and the reduction in IL-1β level was concentration-dependent, with an IC_50_ value (inhibitory effect on IL-1β) of 65.5 ± 7.4 μg/mL (168.6 ± 19.0 μM) (Fig. 4b). Casticin significantly inhibited TNF-α level, with an IC_50_ value (inhibitory effect on TNF-α) of 7.1 ± 0.3 μg/mL (19.0 ± 0.8 μM) (Fig. 4c). Casticin did not have an appreciable effect on IL-1β level at 2 μg/mL; however casticin at 200 μg/mL reduced IL-1β to nearly 50% (Fig. 4d); its IC_50_ value of inhibitory effect on IL-1β could not be determined at the highest concentration of 200 μg/mL tested. Vitexilactone at 20 μg/mL reduced TNF-α level by 30% and a more significant inhibition was observed at higher concentration with an IC_50_ value (inhibitory effect on TNF-α) of 37.5 ± 2.8 μg/mL (99.1 ± 7.4 μM) (Fig. 4e). Similarly, vitexilactone at 20 μg/mL significantly reduced IL1-β level (*p* < 0.05) and further reduction in IL-1β level was observed at higher concentration, with an IC_50_ value (inhibitory effect on IL-1β) of 80.6 ± 9.2 μg/mL (212.9 ± 24.3 μM) (Fig. 4f). Maslinic acid suppressed TNF-α level in a concentration-dependent, with an IC_50_ value (inhibitory effect on TNF-α) of 27.6 ± 1.7 μg/mL (58.4 ± 3.6 μM) (Fig. 4g). On the other hand, maslinic acid significantly increased IL-1β level by 1.7-fold (*p* < 0.05) at 65 μg/mL (Fig. 4h).

**Fig. 5:**
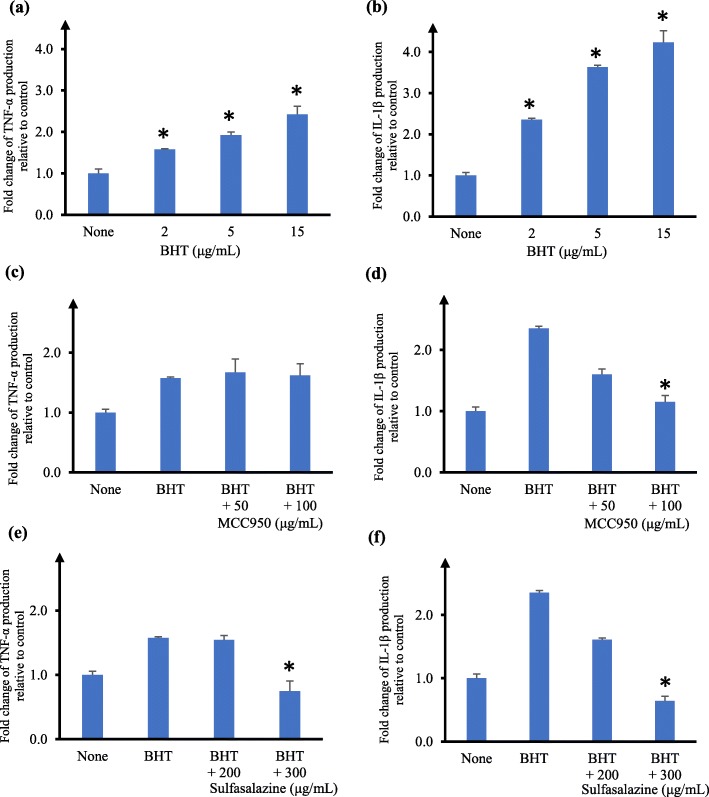
Effects of BHT on TNF-α and IL-1β production by human U937 macrophage cells. Fold change of TNF-α (**a**, **c**, **e**) and IL-1β (**b**, **d**, **f**) production relative to control in the supernatant of human U937 macrophages measured by ELISA. **a**, **b** Cells were treated with none or varying concentrations of butylated hydroxytoluene (BHT) for 18 h. **c-f** Cells were pre-incubated for 6 h with none, 50 μg/mL or 100 μg/mL MCC950 (**c**, **d**), or with 200 μg/mL or 300 μg/mL sulfasalazine (**e**, **f**), followed by stimulation with 2 μg/mL of BHT for 18 h. The concentrations of MCC950 and sulfasalazine used were non-toxic to the cells. Cytokine production by cells not treated with compound (ie. none) was taken as 1. The data are presented as mean fold change ± SD of three independent experiments performed in duplicates. *p < 0.05

We next evaluated the effects of BHT. We observed that cells pre-treated with BHT and then stimulated with LPS did not result in any detectable difference in cytokine level. However, in the absence of LPS stimulation, pre-treatment of human U937 macrophages with BHT increased the production of TNF-α and this increase was concentration-dependent, up to 2.4-fold at 15 μg/mL BHT (Fig. 5a). Similarly, BHT elevated IL-1β production in U937 macrophages and the increase was concentration-dependent, up to 4-fold at 15 μg/mL BHT (Fig. 5b). Subsequent studies on the effects of BHT on TNF-α and IL-1β production in human U937 macrophages were evaluated without LPS stimulation. The inflammasomes are a family of multi-protein cytoplasmic sensors that orchestrate the inflammatory response, of which the NRLP3 inflammasome has been more well characterized [39]. The NLRP3 inflammasome inhibitor MCC950 is known to specifically inhibit IL1-β pathway [40]. We asked if pre-treatment of human U937 macrophages with MCC950 could affect the cytokine level induced by BHT. Addition of MCC950 did not alter TNF-α production in BHT-treated U937 macrophages even up to 100 μg/mL MCC950 (Fig. 5c). There was no observable effect on cell viability to U937 macrophages up to 100 μg/mL MCC950. In contrast, IL-1β production by BHT-treated U937 macrophages was significantly reduced (*p* < 0.05) in the presence of 100 μg/mL MCC950 (Fig. 5d). Therefore, MCC950 reduced IL-1β level but not TNF-α level in BHT-treated U937 macrophages. We next asked if sulfasalazine, a known inhibitor of NF-κB [41], would affect the cytokine level induced by BHT. A significant reduction in TNF-α and IL-1β levels (*p* < 0.05) was observed at 300 μg/mL sulfasalazine (Fig. 5e and f). There was no observable effect on cell viability to U937 macrophages at 200 μg/mL and 300 μg/mL sulfasalazine.

## Discussion

To the best of our knowledge, this is the first study comparing the effects of different types of extraction methods and solvents of *V. trifolia* leaves on cytotoxicity and cytokine production in human U937 macrophages. We found ultrasonication dichloromethane *V. trifolia* leaf extract was comparatively most cytotoxic (IC_50_ 3.2 ± 0.1 μg/mL) while Soxhlet water leaf extract was the least cytotoxic (IC_50_ 684.5 ± 99.0 μg/mL) to U937 macrophages (Table 1). Leaf extracts of both maceration ethanol (Fig. 1c and d) and ultrasonication dichloromethane (Fig. 1e and f) were most active in inhibiting TNF-α and IL-1β levels in U937 macrophages. Previous work has shown Soxhlet methanol-derived *V.trifolia* leaf extract (IC_50_ 6.72 μg/mL) was comparatively less cytotoxic to MCF-7 cells than petroleum-derived leaf extract (IC_50_ 0.41 μg/mL) [42]. In our study, the IC_50_ of Soxhlet methanol *V.trifolia* leaf extract in U937 macrophage cells was 145.1 ± 13.7 μg/mL, which is comparatively less toxic than that reported by Garbi et al [42]. Vasanthi and colleagues reported cytotoxicity of Soxhlet hexane *V. trifolia* leaf extract in MCF-7 and HeLa cells, with both showing an IC_50_ of 80 μg/mL [43]. We found that the IC_50_ of Soxhlet hexane *V. trifolia* leaf extract in U937 macrophages was 5.2 ± 0.5 μg/mL (Table 1), which is comparatively more toxic than that reported by Vasanthi et al [43]. These differences may be due to the different cell lines studied. Separately, cytotoxic activities of *V. trifolia* aerial extracts in methanol, ethyl acetate and chloroform were evaluated using brine shrimp bioassay method, and the LC_50_ values were 140 mg/mL, 165 mg/mL and 180 mg/mL, respectively [44]. These LC_50_ values were much higher than the IC_50_ values we observed in our study (Table 1), most likely due to the different assays used. Kumar-Roiné et al [10] showed aqueous decoction of *V.trifolia* leaves inhibited nitric oxide at IC_50_ of 13.8 mg/mL extract in RAW264.7 murine macrophages, and the aqueous extract had no significant toxicity in LPS-stimulated RAW264.7. In another study, 2.5 mg/mL aqueous leaf extract of *V.trifolia* suppressed significantly the mRNA production of LPS-induced chemokines C-X-C motif 10 (CXCL-10), C–C motif ligand 3(CCL-3) and cyclo-oxygenase (COX)-2 [23]. Further, 2.5 mg/mL aqueous leaf extract significantly inhibited mRNA production of IL-1β, IL-6, TNF-α and iNOS, elevated IL-10 mRNA, and reduced the protein levels of IL-6 (67.5% inhibition) and TNF-α (10.4% inhibition), and increased IL-10 protein level (3.5-fold) [11]. In contrast to Matsui et al [11], we observed a more pronounced reduction in TNF-α protein in U937 macrophages by maceration ethanol leaf extract (Fig. 2a) and ultrasonication dichloromethane leaf extract (Fig. 2c). This may be due to the different experimental systems used. We noted that our Soxhlet water leaf extract at 100 μg/mL did not result in any significant reduction in TNF-α protein level in U937 macrophages (Fig. 1c). This is in agreement with the observations in murine macrophages by Matsui et al [11].

Further investigation of our most active leaf extracts (ie. maceration ethanol and ultrasonication dichloromethane leaf extracts) led to the isolation and identification of artemetin, casticin, vitexilactone and maslinic acid. In total, 12 compounds were identified in the *V. trifolia* leaf extracts (Fig. 3). To the best of our knowledge, this is the first report of BHT, 2,4-di-*tert*-butylphenol, campesterol and maslinic acid in the leaf extracts of *V. trifolia*. Artemetin and casticin (Fig. 3) have been identified from ethanolic extracts of dried fruits of *V. trifolia* [45, 46] and methanolic extracts of dried leaves and twigs of *V. trifolia* [17]. Both artemetin and casticin are reported to have potent lipoxygenase inhibition, with casticin two times more potent than artemetin [47]. Artemetin is shown to have anti-inflammatory activity using various experimental models in rats, including inhibiting carrageenan-induced paw edema, reduced granuloma formation and reduced vascular permeability to intracutaneous histamine [48]. Artemetin can also protect endothelial function by acting as an anti-oxidant and anti-apoptotic agent [49]. We showed that artemetin inhibited the production of both TNF-α and IL-1β (Fig. 4a and b). To the best of our knowledge, this is the first report that shows artemetin can inhibit TNF-α and IL-1β cytokine production in human U937 macrophages. We observed that artemetin inhibited cell viability of U937 macrophages, consistent with several reports showing artemetin inhibited cell viability. Ono et al [50] reported that artemetin showed a GI_50_ of 2270 ng/mL in human lung cancer PC-12 cells and 2200 ng/mL in human colon cancer HCT116 cells. Artemetin decreased growth of human leukemia HL-60 cells in dose-dependent manner, with IC_50_ of 39.98 μM after 96 h [51]. Casticin is reported to alleviate airway inflammation by suppressing pro-inflammatory cytokine production such as TNF-α in the lungs and bronchoalveolar lavage fluid in an inflammatory murine model of asthma [52]. Casticin inhibited TNF-α and IL-1β cytokine production in LPS-stimulated mouse macrophages at the range of 3 μM to 10 μM of casticin [53]. Our results showing casticin inhibited TNF-α production (Fig. 4c) are in the similar range reported by Liou et al [53]. Interestingly, we observed that the inhibitory effects of casticin on IL-1β cytokine production in human U937 macrophages were about 100-fold higher (Fig. 4d) than that reported by Liou et al [53]. This may be attributed to the different experimental systems. The molecular mechanism responsible for the anti-inflammatory activity of casticin likely involved NF-κB, AKT and MAPK signaling pathways [53]. In human umbilical vein endothelial cells, casticin significantly decreased vascular inflammation through inhibiting ROS-NF-κB pathway [54].

Vitexilactone, a labdane-type diterpenoid (Fig. 3), has been isolated from the fruits of *V. trifolia* and *V. agnus-castus* [55, 56]. It was reported to induce adipogenesis in 3T3-L1 preadipocytes [57]. Fang and colleagues showed that in HEK293 cell line, vitexilactone D inhibited TNF-α induced NF-κB activation [58]. In our study, we found vitexilactone inhibited the production of TNF-α and IL-1β (Fig. 4e and f). We did not observe any appreciable cytotoxicity of vitexilactone in human U937 macrophages, similar to a report indicating vitexilactone up to 100 μM had negligible cytotoxicity in mouse 3T3-L1 preadipocytes [57]. The phytoconsitutents β-sitosterol, campesterol, stigmasterol and phytol (Fig. 3) identified in our leaf extracts are known to have anti-inflammatory properties amongst other biological activities, such as anti-oxidant and anti-angiogenic [59, 60]. Both β-sitosterol and stigmasterol have been previously isolated in *V. trifolia* leaf extracts [61]. As far as we are aware, this is the first report of campesterol in *V. trifolia* leaf extracts. Campesterol is found in other *Vitex* species, such as leaves of *V. agnus-castus* [62].

Maslinic acid, an oleanane-type triterpenoid (Fig. 3), has been isolated from *V. negundo*, *V. altissima*, and *V. agnus-castus* [63]. Maslinic acid is reported to reduce neuroinflammation in cultured rat cortical astrocytes by inhibiting nitric oxide and TNF-α mRNA and protein levels through NF-κB signaling pathway [64]. The secretion of the inflammatory cytokines IL-6 and TNF-α from LPS-stimulated murine macrophages were significantly reduced (*p* < 0.01) by 50 μM and 100 μM of maslinic acid [65]. In THP-1 cells, maslinic acid enhanced the recruitment of macrophages by elevating the production of IL-8, IL-1α, and IL-1β [66]. Maslinic acid suppressed TNF-α production in RAW264.7 cells, and maslinic acid had anti-inflammatory effects in carrageenan-induced paw edema model, as well as anti-arthritis effects in mice models of arthritis [67]. In our study, maslinic acid suppressed TNF-α and enhanced IL-1β in human U937 macrophages (Fig. 4g and h), which is in alignment with observations by others [64–67]. The other two terpenoids identified in our study, α-amyrin and β-amyrin (Fig. 3), are known to have anti-inflammatory activities [68], and have been previously reported in *V. trifolia* leaves [16].

The phenolic compound 2,4 di-*tert*-butylphenol (Fig. 3) is typically used as an intermediate in preparing UV stabilizers and antioxidants, and in the manufacture of pharmaceuticals and fragrances [69]. It is found naturally occurring in nature, for example in lactic acid bacteria *Lactococcus* sp. [70], leaves of *Pereskia bleo* (Kunth) [71], and roots of *Humboldtia unijuga* [72]. To the best of our knowledge, this is the first report of 2,4-di-*tert*-butylphenol in *V. trifolia* leaf extracts. Besides its antioxidant property, 2,4 di-*tert*-butylphenol also has antifungal, antitumor activities and anti-inflammatory activities [70, 72]. Another naturally occurring phenolic compound is BHT (Fig. 3), which can be found in freshwater phytoplankton, including a green alga and three cyanobacteria [73], and plants such as *Cytisus triflorus* [74], *Mesembryanthemum crystallinum* [75] and seeds of *Trichilia emetic* commonly known as natal mahogany [76]. Originally found as a synthetic antioxidant, BHT has been extensively used in food industry, petroleum products and rubber [76]. Typically used as food preservative, BHT has been restricted in its use as it may be toxic at higher concentrations; indeed, BHT is applied as an inducer for animal lung tumor models [77]. To the best of our knowledge, this is the first report of BHT in *V. trifolia* leaf extracts. Our findings that BHT increased TNF-α and IL-1β cytokine production in human U937 macrophages (Fig. 5) suggest BHT exert pro-inflammatory effects. To the best of our knowledge, this is the first report showing the pro-inflammatory effects of BHT on TNF-α and IL-1β protein levels in human U937 macrophages. Murakami et al [78] studied LPS-stimulated murine macrophage RAW264.7 cells treated with BHT and did not find any significant difference in TNF-α mRNA expression compared with non-treated control cells. Their study on BHT was conducted in the presence of LPS, in contrast to our study performed in the absence of LPS, which could account for the difference in observations. The presence of LPS likely masked any increase in cytokine signal elicited by BHT; indeed, we noted that cells pre-treated with BHT and then stimulated with LPS did not result in any alteration in cytokine level. Our results along with other published literature point to the pro-inflammatory effects of BHT. Increased cyclooxygenase-1 and cyclooxygenase-2 expression, and increased inflammatory cell infiltration were observed in lung tumor formation in BALB mice models caused by BHT administration following an initiating agent [77]. There was elevated production of pro-inflammatory mediators such as prostaglandin, and increased translocation of 5-lipoxygenase from the cytosol to the membrane which could be partially inhibited by celecoxib, an inhibitor of cyclooxygenase-2 enzyme [79]. Future work will include using in vivo models such as a carrageenan-induced paw-edema model to validate the activity of the individual compounds identified. Further, the physiological significance of these individual compounds may be evaluated in monocytes freshly extracted from rats, and performing western blot analyses.

## Conclusions

In conclusion, leaf extracts of *V. trifolia* obtained using different solvents and extraction methods were successfully investigated for their effects on cytokine production in human U937 macrophages. The findings provide scientific evidence for the traditional use of *V. trifolia* leaves (a sustainable resource) and highlight the importance of conservation of medicinal plants as resources for drug discovery. Our results together with others suggest further investigation on *V. trifolia* and constituents to develop novel treatment strategies in immune-mediated inflammatory conditions is warranted.

## Data Availability

Data are available on request due to privacy or other restrictions.

## Abbreviations

BHT: Butylated hydroxytoluene
Dex: Dexamethasone
DCM: Dichloromethane
DMSO: Dimethyl sulfoxide
ELISA: Enzyme-linked immunosorbent assay
GC-MS: Gas chromatography-mass spectrometry
HPLC: High performance liquid chromatography
IC_50_: Inhibitory concentration required for 50% inhibition of activity
IC_20_: Inhibitory concentration required for 20% inhibition of activity
IL-1β: Interleukin-1β
LC-MS: Liquid chromatography-mass spectrometry
LPS: Lipopolysaccharide
Mac: Maceration
NLRP3: Nod-like receptor protein family pyrin domain containing 3
PBS: Phosphate buffered saline
PMA: Phorbol 12-myristate 13-acetate
Sox: Soxhlet
TNF-α: Tumor necrosis factor-α
US: Ultrasonication
WST-1: Water soluble tetrazolium salt
